# Improving quality by improving safety

**Published:** 2014

**Authors:** Rachel Mearkle, Dan Bwonya, Robert Lindfield

**Affiliations:** Speciality Registrar: Public Health Medicine, International Centre for Eye Health, London School of Hygiene and Tropical Medicine, London, UK; Ophthalmologist: Mengo Hospital, Kampala, Uganda.; Lecturer: International Centre for Eye Health and Advisor: ORBIS, London, UK. Robert.Lindfield@Lshtm.ac.uk

**Figure:**
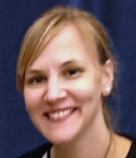
Rachel Mearkle

**Figure:**
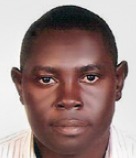
Dan Bwonya

**Figure:**
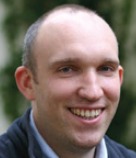
Robert Lindfield

Protecting patients from harm is one of the most important things health professionals can do for their patients. However, an estimated 1 in 10 patients are harmed while receiving hospital care in high-income countries,^1^ and studies suggest that the rates of harm are higher in low- and middle-income countries.^2^ The World Health Organization (WHO) describes safety as a key dimension of quality health care.^3^

Patient safety means ‘the absence of preventable harm to a patient during the process of health care.’^1^ It includes issues such as the prevention of hospital-acquired infections, surgical safety, medication errors, falls and accidents in hospital.

## Keeping patients safe in theatres

Keeping patients safe before, during and after operations is vital. Simple, cheap interventions can be implemented to prevent harm in theatres. The WHO has developed a surgical safety checklist^4^ to be used for each operation; use of the checklist can reduce the rate of post-operative deaths and complications by more than one third. Evidence suggests that this tool may be particularly effective in low-income settings.^5^ A surgical safety checklist is simple, quick and can be adapted to suit local needs. For example, a specific checklist for cataract surgery has been developed in the UK.^6^

### What you can do

- Develop a local surgical safety checklist, using the WHO tool asa model, which you can use on every surgical patient to prevent errors in theatre.
- Ensure surgeons perform a ‘double check’ (see panel) on every patient to check they have the correct patient, correct eye and correct equipment; this will highlight any errors before the operation takes place.

**The theatre ‘double check’**

Before each operation, the surgeon must do the following checks.

- **Correct patient.** Check the patient's name and date of birth with them, and check you have the correct notes.
- **Correct eye.** Check with the patient which eye you are operating on and check this in the patient's notes.
- **Correct IOL.** Check with the theatre assistant, and in the patient notes, to ensure that you have the correct intra-ocular lens (IOL).

### Protecting patients from infection

Infections acquired in hospital are a major global problem. In low-income countries, an estimated 15.5 out of every 100 patients treated will develop an infection asa result of their contact with the health service. Clean hands are an important step in preventing transmission of infections. Hand hygiene measures can reduce the frequency of health care associated infections by more than 50%.^1^

Eye infection can spread in hospitals where infection control measures are inadequate. In eye units, inadequate access to working sinks, hand towels, and alcohol hand rubs can make effective hand hygiene difficult.

### What you can do

- Always clean your hands between each patient, either with soap and water or using alcohol hand rub.
- Make hospital management aware if hand hygiene facilities need to be improved. It should be easy for you to wash your hands anywhere that you are seeing patients.

**Figure:**
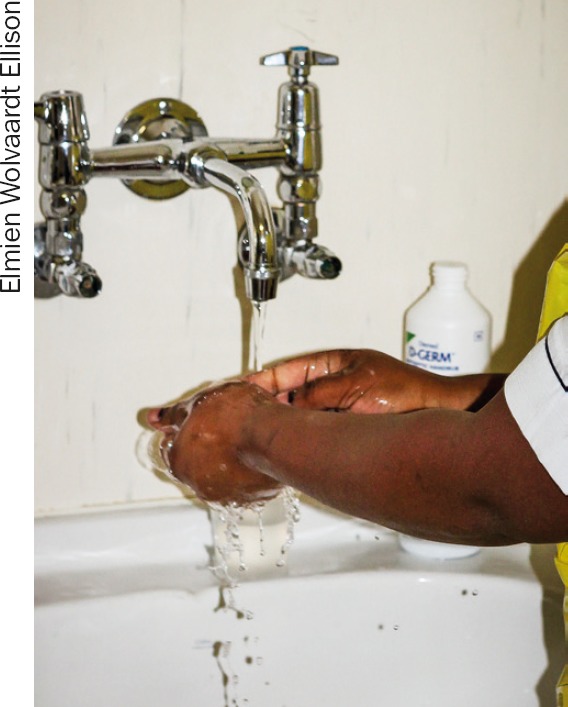
Hand hygiene measures can reduce health care associated infections by 50%

## Learn from mistakes

Accidents and ‘near misses’ (potential accidents that have been prevented just in time) happen in every health system in the world, and evidence shows that the majority of errors in healthcare are preventable. It is vital that we learn from mistakes and near misses so that they are not repeated. It is also important to ask patients about their experiences in hospital in order to identify areas that could be improved. For example, when discharging a patient it is important that they know how to care for themselves at home; however, this is often not explained well. You can assess how well patients have understood by asking the patient to tell you how they need to care for their eye at home.

### What you can do

- When mistakes happen, report them, find the cause of the mistake and take steps to prevent a similar situation in the future.
- Ask patients about their experiences in hospital and act on the information they tell you.

Keeping patients safe is a vital component of providing a high quality service. There are very simple, inexpensive steps that can be taken to dramatically improve safety in your hospital. Eye care cannot be considered high quality without taking these basic measures to keep patients safe.

**Case study: Ruharo Eye Centre**

In 2013 Ruharo Eye Centre in Uganda participated in a study of hospital quality. As a result of the study the hospital conducted several continuous medical education sessions for its staff; the sessions re-emphasised a culture of hand washing before and after contact with patients. After the sessions the hospital started receiving information in the suggestion box thanking the hospital for the high level of hygiene experienced by patients while at the hospital. This improved patient management and the hospital's image in the community.
